# Supplementary material for: Changes in contraceptive and sexual behaviours among unmarried young people in Nigeria: Evidence from nationally representative surveys
Source: PLoS One. 2021 Feb 2;16(2):e0246309. doi: 10.1371/journal.pone.0246309 (PMC7853509; doi:10.1371/journal.pone.0246309)
Supplement: Attachment — Submitted filename: Response to Editors comment.docx [file pone.0246309.s002.docx]

**Step-by-step responses to the comments**

**Comments**

Religion has been added to the analysis but not to table 1.

**Response**

We have included religion in Table 1 with the corresponding operational definition (Religious affiliation of the respondents) and grouping for analytical purpose ((1) Christianity; (2) Islam; (3) Traditional and others)

**Comment**

Avoid the use of the word “respondents” in discussing table 2, since you are not talking about the characteristics of the survey respondents: the biased characteristics depend on the selection strategy:  never married sexually active. That has to be born in mind in the interpretation of results, which currently is not (lines 174), which should be rewritten after redesign as suggested below.

- As reviewer 1 originally pointed out, this is important for interpretation. The current design of table 2 incorporating the proportion only for the age-group with a different format and a footnote is not satisfactory. Reading the table one wonders why there is an overrepresentation of wealthier / more educated. It could be because of sexual activity or because of selection in marriage, and those go in different directions. Please redesign the table in the following way:
  - Omit absolute numbers, except in the header (as it currently stands)
  - Provide nine columns, three for each survey, including percentages by socidemographic characteristics for the three subgroups of married, not sexually active, and unmarried sexually active (your group of interest). You could include also a chi-square test of association if desired.
  - The interpretation for the first part of results becomes then much more focused: most likely the over representation of wealthier/more educated will be due to lower prevalence of marriage, but there might also be differences in sexual activity. Just rephrase.

**Response**

The result in Table 2 has been revised and interpreted as advised

**Comments**

Line 118: Remove “been” should be “to have ever engaged in sexual intercourse”

**Response**

The word “been” in Line 118 has been removed from the sentence which is now revised as “to have ever engaged in sexual intercourse”

**Comments**

Line 445, 449 highlight higher risk for female, but this is not true for all dimensions (multiple partners goes in the different direction). Please rephrase so that it leads naturally to lines 451-452 saying that SRH policies need to “account for specific gender peculiarities”.

**Response**

The sentences in Line 445 and 449 have been rephrased and expanded.

**Comments**

Rephrase line 80 as “and of the adverse consequences of unmet need among young people such as”

**Response**

The sentence in line 80 has been rephrased as suggested by the reviewer
